# 1-Piperonylpiperazinium 4-nitro­benzoate monohydrate

**DOI:** 10.1107/S160053681400261X

**Published:** 2014-02-12

**Authors:** Channappa N. Kavitha, Manpreet Kaur, Brian J. Anderson, Jerry P. Jasinski, H. S. Yathirajan

**Affiliations:** a Department of Studies in Chemistry, University of Mysore, Manasagangotri, Mysore 570 006, India; b Department of Chemistry, Keene State College, 229 Main Street, Keene, NH 03435-2001, USA

## Abstract

In the title hydrated salt [systematic name: 1-(1,3-benzodioxol-5-ylmeth­yl)piperazin-1-ium 4-nitro­benzoate monohydrate], C_12_H_17_N_2_O_2_^+^·C_7_H_4_NO_4_^−^·H_2_O, the piperazinium ring of the cation adopts a slightly distorted chair conformation. The piperonyl and piperazine rings are rotated with respect to each other with an N—C—C—C torsion angle of 45.6 (2)°. In the anion, the nitro group is almost coplanar with the adjacent benzene ring, forming a dihedral angle of only 3.9 (4)°. In the crystal, the cations, anions and water mol­ecules are linked through N—H⋯O and O—H⋯O hydrogen bonds into chains along the *a* axis. In addition, weaker inter­molecular C—H⋯O inter­actions are also observed within the chains. The anions form centrosymmetric couples through π-stacking inter­actions, with an inter­centroid distance of 3.681 (4) Å between the benzene rings.

## Related literature   

For the drug, piribedil {systematic name: 2-[4-(benzo[1,3]dioxol-5-ylmeth­yl)piperazin-1-yl]pyrimidine}, an anti­parkin­sonian agent, see: Millan *et al.* (2001 ▶). For piperonylpiperazine derivatives with α-adrenergic antagonist and vasodilator properties, see: Gobert *et al.* (2003 ▶); Gilbert *et al.* (1968 ▶). For the use of piperazine in the construction of various bioactive mol­ecules, see: Choudhary *et al.* (2006 ▶). For the anti­microbial activity of piperazine derivatives, see: Kharb *et al.* (2012 ▶). For related biologically active compounds, see: Brockunier *et al.* (2004 ▶); Bogatcheva *et al.* (2006 ▶). For a review on the current pharmacological and toxicological information for piperazine derivatives, see: Elliott (2011 ▶). For a related structure, see: Capuano *et al.* (2000 ▶). For puckering parameters, see Cremer & Pople (1975 ▶). For standard bond lengths, see: Allen *et al.* (1987 ▶).
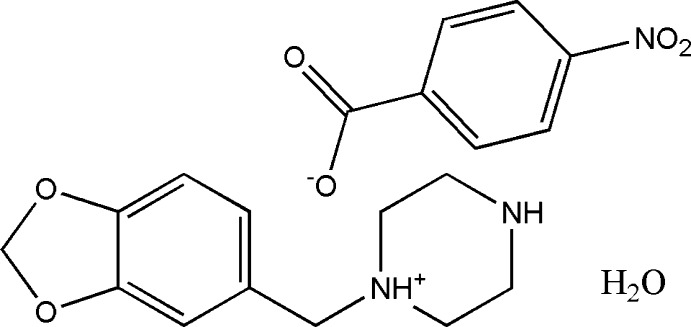


## Experimental   

### 

#### Crystal data  

C_12_H_17_N_2_O_2_^+^·C_7_H_4_NO_4_^−^·H_2_O
*M* *_r_* = 405.40
Triclinic, 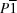

*a* = 6.0745 (5) Å
*b* = 12.0617 (11) Å
*c* = 13.4817 (10) Å
α = 92.561 (7)°
β = 98.753 (7)°
γ = 93.326 (7)°
*V* = 973.20 (14) Å^3^
*Z* = 2
Cu *K*α radiation
μ = 0.90 mm^−1^
*T* = 173 K
0.42 × 0.36 × 0.24 mm

#### Data collection  

Agilent Xcalibur (Eos, Gemini) diffractometer
Absorption correction: multi-scan (*CrysAlis PRO* and *CrysAlis RED*; Agilent, 2012 ▶) *T*_min_ = 0.882, *T*_max_ = 1.000
6403 measured reflections
3761 independent reflections
3196 reflections with *I* > 2σ(*I*)
*R*_int_ = 0.021

#### Refinement  

*R*[*F* ^2^ > 2σ(*F* ^2^)] = 0.043
*wR*(*F* ^2^) = 0.120
*S* = 1.03
3761 reflections
263 parameters
H-atom parameters constrained
Δρ_max_ = 0.27 e Å^−3^
Δρ_min_ = −0.20 e Å^−3^

### 

Data collection: *CrysAlis PRO* (Agilent, 2012 ▶); cell refinement: *CrysAlis PRO*; data reduction: *CrysAlis RED* (Agilent, 2012 ▶); program(s) used to solve structure: *SUPERFLIP* (Palatinus & Chapuis, 2007 ▶; Palatinus & van der Lee, 2008 ▶; Palatinus *et al.*, 2012 ▶); program(s) used to refine structure: *SHELXL97* (Sheldrick, 2008 ▶); molecular graphics: *OLEX2* (Dolomanov *et al.*, 2009 ▶); software used to prepare material for publication: *OLEX2*.

## Supplementary Material

Crystal structure: contains datablock(s) I. DOI: 10.1107/S160053681400261X/ld2118sup1.cif

Structure factors: contains datablock(s) I. DOI: 10.1107/S160053681400261X/ld2118Isup2.hkl

Supporting information file. DOI: 10.1107/S160053681400261X/ld2118Isup3.cml

CCDC reference:

Additional supporting information:  crystallographic information; 3D view; checkCIF report

## Figures and Tables

**Table 1:** Hydrogen-bond geometry (Å, °)

*D*—H⋯*A*	*D*—H	H⋯*A*	*D*⋯*A*	*D*—H⋯*A*
N2*A*—H2*AA*⋯O1*W* ^i^	0.94	1.84	2.7800 (16)	172
N2*A*—H2*AB*⋯O1*B* ^ii^	0.93	1.80	2.7262 (16)	175
C9*A*—H9*AA*⋯O2*A* ^iii^	0.99	2.58	3.3260 (19)	132
C10*A*—H10*A*⋯O1*W* ^iv^	0.99	2.51	3.2833 (19)	135
O1*W*—H1*WA*⋯O2*B* ^v^	0.90	1.76	2.6526 (16)	170
O1*W*—H1*WB*⋯O1*B* ^ii^	0.92	1.90	2.7867 (16)	163

Symmetry codes: (i) 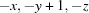; (ii) 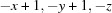; (iii) 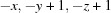; (iv) 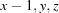; (v) 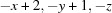.

**Fig. 1.**
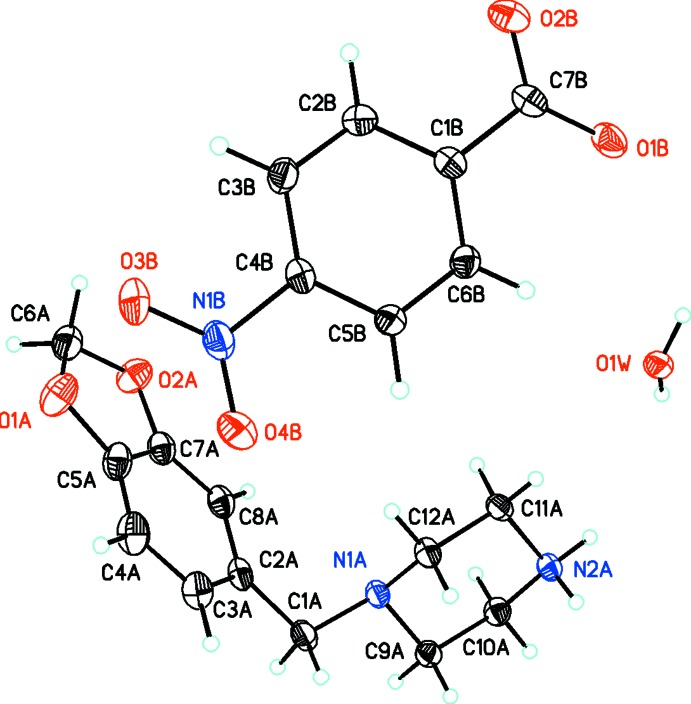
ORTEP drawing of one independent monocation-monoanion-water molecule unit in the asymmetric unit of (I) (C12H17N2O2+ . C7H4NO4- . H2O) showing the labeling scheme with 30% probability displacement ellipsoids.

**Fig. 2.**
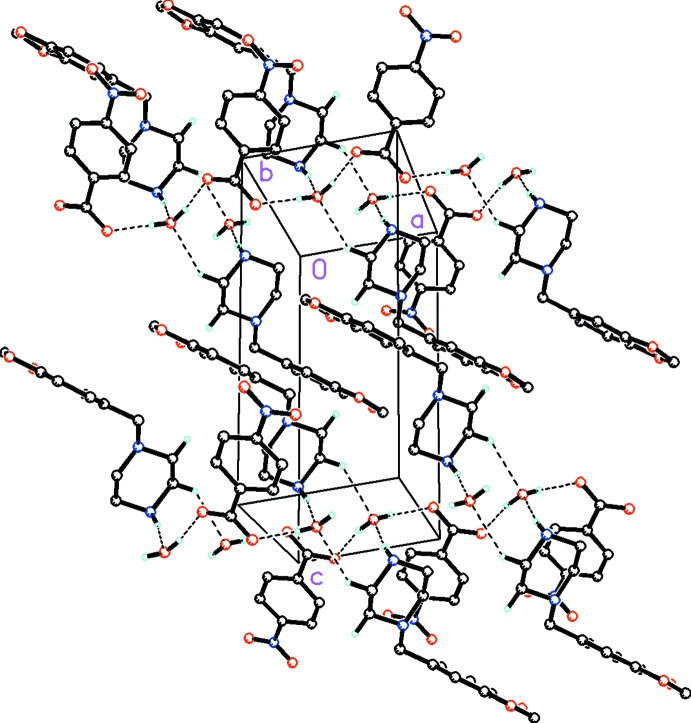
Molecular packing for (I) viewed along the b axis. Dashed lines indicate N—H···O, O—H···O intermolecular hydrogen bonds and weak C—H···O intermolecular interactions. H atoms not involved in hydrogen bonding have been removed for clarity.

## supplementary crystallographic information

### 1. Comment

1-(3,4-Methylenedioxybenzyl)piperazine or 1-piperonylpiperazine is a psychoactive drug of the piperazine class and is used to synthesise the drug, piribedil, an antiparkinsonian agent (Millan *et al.*, 2001). Piperonylpiperazine derivatives also has α-adrenergic antagonist properties (Gobert *et al.*, 2003) and peripheral vasodilator properties (Gilbert *et al.*, 1968). The piperazine moiety is extensively employed to construct various bioactive molecules with anti-bacterial, antimalarial activity and as antipsychotic agents (Choudhary *et al.*, 2006). A valuable insight into recent advances on antimicrobial activity of piperazine derivatives is reported (Kharb *et al.*, 2012). Piperazines are among the most important building blocks in today's drug discovery and are found in biologically active compounds across a number of different therapeutic areas (Brockunier *et al.*, 2004; Bogatcheva *et al.*, 2006). A review on the current pharmacological and toxicological information for piperazine derivatives is available (Elliott, 2011). The crystal structure of an N-piperonyl analogue of the atypical antipsychotic clozapine (Capuano *et al.*, 2000) is reported. In continuation of our work on salts of piperonylpiperazines, this paper reports the crystal structure of the title compound, (I), C_12_H_17_N_2_O_2_^+^ . C_7_H_4_NO_4_^-^ . H_2_O.

The asymmetric unit of the title compound, (I), contains one independent 1-piperonylpiperazinium monocation, one 4-nitrobenzoate monoanion and one water molecule (Fig. 1). The piperazine ring in the cation adopts a slightly disordered chair conformation (puckering parameters Q, θ, and φ = 0.590 (2)Å, 3.8 (6)° and 1.68 (4)°; (Cremer & Pople, 1975). The piperonyl and piperazine rings are twisted with respect to each other with an N1A/C1A/C2A/C8A torsion angle of 45.6 (2)°. In the anion, the nitro substituent is slightly twisted from the mean plane of the phenyl ring with a dihedral angle of 3.9 (4)°. Bond lengths are in normal ranges (Allen *et al.*, 1987). In the crystal, the cations and anions interact through N—H···O intermolecular hydrogen bonds while weak C—H···O intermolecular interactions are observed between the cations (Fig. 2). The crystal packing is stabilized by these N—H···O and O—H···O intermolecular hydrogen bonds and weak C—H···O intermolecular interactions (Table 1) involving the water molecules which form 1D chains along [1 0 0]. In addition, weak Cg5–Cg5 π–π stacking interactions with an intercentroid distance of 3.681 (4)Å (Symmetry operation 2-x, -y, -z; Cg5 is the centroid between the phenyl rings, C1B–C6B, of the anions) contribute to the crystal packing.

### 2. Experimental

1-piperonylpiperazine ( 2.2g, 0.01 mol) and p-nitrobenzoic acid (1.67 g, 0.01 mol) were dissolved in hot N,N-dimethylformamide and stirred for 10 mins at 323 K. The resulting solution was allowed to cool slowly at room temperature. The crystals of the title salt appeared after a few days was used as such for x-ray studies (m. p:448-451 K).

### 3. Refinement

All of the H atoms were placed in their calculated positions and then refined using the riding model with Atom—H lengths of 0.95Å (CH) , 0.99Å (CH_2_), 0.92 or 0.94Å (NH_2_), 0.89 or 0.91Å (OH_2_). Isotropic displacement parameters for these atoms were set to 1.2 (CH, CH_2_, NH_2_) or 1.5 (OH_2_) times *U*_eq_ of the parent atom.

### Crystal data

**Table:**

C_12_H_17_N_2_O_2_^+^·C_7_H_4_NO_4_^−^·H_2_O	*Z* = 2
*M**_r_* = 405.40	*F*(000) = 428
Triclinic, *P*1	*D*_x_ = 1.383 Mg m^−^^3^
*a* = 6.0745 (5) Å	Cu *K*α radiation, λ = 1.54184 Å
*b* = 12.0617 (11) Å	Cell parameters from 2866 reflections
*c* = 13.4817 (10) Å	θ = 3.3–72.4°
α = 92.561 (7)°	µ = 0.90 mm^−^^1^
β = 98.753 (7)°	*T* = 173 K
γ = 93.326 (7)°	Irregular, colourless
*V* = 973.20 (14) Å^3^	0.42 × 0.36 × 0.24 mm

### Data collection

**Table:**

Agilent Xcalibur (Eos, Gemini) diffractometer	3761 independent reflections
Radiation source: Enhance (Cu) X-ray Source	3196 reflections with *I* > 2σ(*I*)
Detector resolution: 16.0416 pixels mm^-1^	*R*_int_ = 0.021
ω scans	θ_max_ = 72.4°, θ_min_ = 3.3°
Absorption correction: multi-scan (*CrysAlis PRO* and *CrysAlis RED*; Agilent, 2012)	*h* = −7→7
*T*_min_ = 0.882, *T*_max_ = 1.000	*k* = −14→13
6403 measured reflections	*l* = −16→15

### Refinement

**Table:**

Refinement on *F*^2^	Hydrogen site location: mixed
Least-squares matrix: full	H-atom parameters constrained
*R*[*F*^2^ > 2σ(*F*^2^)] = 0.043	*w* = 1/[σ^2^(*F*_o_^2^) + (0.0563*P*)^2^ + 0.0984*P*] where *P* = (*F*_o_^2^ + 2*F*_c_^2^)/3
*wR*(*F*^2^) = 0.120	(Δ/σ)_max_ < 0.001
*S* = 1.03	Δρ_max_ = 0.27 e Å^−^^3^
3761 reflections	Δρ_min_ = −0.20 e Å^−^^3^
263 parameters	Extinction correction: *SHELXL97* (Sheldrick, 2008), Fc^*^=kFc[1+0.001xFc^2^λ^3^/sin(2θ)]^-1/4^
0 restraints	Extinction coefficient: 0.0049 (6)
Primary atom site location: structure-invariant direct methods	

### Special details

**Table:**

Geometry. All esds (except the esd in the dihedral angle between two l.s. planes) are estimated using the full covariance matrix. The cell esds are taken into account individually in the estimation of esds in distances, angles and torsion angles; correlations between esds in cell parameters are only used when they are defined by crystal symmetry. An approximate (isotropic) treatment of cell esds is used for estimating esds involving l.s. planes.

### Fractional atomic coordinates and isotropic or equivalent isotropic displacement parameters (Å^2^)

**Table:**

	*x*	*y*	*z*	*U*_iso_*/*U*_eq_	
O1A	0.5807 (2)	0.15502 (11)	0.58196 (11)	0.0590 (4)	
O2A	0.5429 (2)	0.34467 (10)	0.59022 (10)	0.0501 (3)	
N1A	−0.1474 (2)	0.41016 (10)	0.31368 (9)	0.0318 (3)	
N2A	−0.1698 (2)	0.54167 (10)	0.14110 (9)	0.0332 (3)	
H2AA	−0.2433	0.4918	0.0891	0.040*	
H2AB	−0.1248	0.6053	0.1115	0.040*	
C1A	−0.1964 (3)	0.31162 (14)	0.36785 (13)	0.0403 (4)	
H1AA	−0.2762	0.2532	0.3195	0.048*	
H1AB	−0.2965	0.3308	0.4166	0.048*	
C2A	0.0109 (3)	0.26602 (13)	0.42328 (11)	0.0369 (3)	
C3A	0.0366 (3)	0.15269 (14)	0.41979 (13)	0.0446 (4)	
H3A	−0.0772	0.1047	0.3811	0.054*	
C4A	0.2234 (3)	0.10638 (14)	0.47100 (14)	0.0500 (4)	
H4A	0.2393	0.0285	0.4678	0.060*	
C5A	0.3822 (3)	0.17849 (14)	0.52603 (12)	0.0426 (4)	
C6A	0.6796 (3)	0.25825 (15)	0.62731 (13)	0.0466 (4)	
H6AA	0.8318	0.2712	0.6106	0.056*	
H6AB	0.6907	0.2575	0.7013	0.056*	
C7A	0.3587 (3)	0.29153 (13)	0.53067 (11)	0.0375 (3)	
C8A	0.1769 (3)	0.33840 (13)	0.48093 (12)	0.0378 (3)	
H8A	0.1632	0.4165	0.4851	0.045*	
C9A	−0.3555 (2)	0.45704 (13)	0.27135 (11)	0.0337 (3)	
H9AA	−0.4451	0.4722	0.3254	0.040*	
H9AB	−0.4436	0.4028	0.2212	0.040*	
C10A	−0.3064 (3)	0.56353 (13)	0.22182 (12)	0.0353 (3)	
H10A	−0.4481	0.5943	0.1927	0.042*	
H10B	−0.2245	0.6190	0.2726	0.042*	
C11A	0.0375 (2)	0.48756 (13)	0.18124 (12)	0.0349 (3)	
H11A	0.1350	0.5397	0.2300	0.042*	
H11B	0.1200	0.4685	0.1256	0.042*	
C12A	−0.0217 (3)	0.38327 (12)	0.23225 (11)	0.0336 (3)	
H12A	−0.1124	0.3295	0.1826	0.040*	
H12B	0.1165	0.3483	0.2598	0.040*	
O1B	1.02663 (19)	0.27812 (9)	−0.04752 (9)	0.0437 (3)	
O2B	1.3475 (2)	0.26959 (12)	0.05605 (11)	0.0591 (4)	
O3B	0.8956 (2)	−0.16582 (12)	0.28622 (10)	0.0616 (4)	
O4B	0.5845 (2)	−0.15818 (12)	0.18715 (11)	0.0595 (4)	
N1B	0.7773 (2)	−0.12428 (11)	0.21838 (10)	0.0409 (3)	
C1B	1.0487 (2)	0.14427 (11)	0.07692 (11)	0.0301 (3)	
C2B	1.1819 (2)	0.09318 (13)	0.15270 (12)	0.0357 (3)	
H2B	1.3335	0.1195	0.1723	0.043*	
C3B	1.0959 (3)	0.00431 (13)	0.19982 (12)	0.0369 (3)	
H3B	1.1868	−0.0319	0.2505	0.044*	
C4B	0.8739 (2)	−0.02983 (12)	0.17066 (11)	0.0319 (3)	
C5B	0.7362 (2)	0.02062 (12)	0.09760 (11)	0.0329 (3)	
H5B	0.5832	−0.0040	0.0803	0.039*	
C6B	0.8256 (2)	0.10799 (12)	0.04996 (11)	0.0329 (3)	
H6B	0.7341	0.1431	−0.0013	0.039*	
C7B	1.1503 (3)	0.23837 (12)	0.02427 (12)	0.0355 (3)	
O1W	0.34602 (17)	0.60766 (9)	0.01811 (8)	0.0378 (3)	
H1WA	0.4609	0.6478	−0.0004	0.057*	
H1WB	0.2443	0.6571	0.0317	0.057*	

### Atomic displacement parameters (Å^2^)

**Table:**

	*U*^11^	*U*^22^	*U*^33^	*U*^12^	*U*^13^	*U*^23^
O1A	0.0637 (8)	0.0486 (8)	0.0625 (8)	0.0261 (6)	−0.0051 (7)	−0.0006 (6)
O2A	0.0471 (7)	0.0435 (7)	0.0574 (8)	0.0115 (5)	−0.0010 (6)	−0.0032 (6)
N1A	0.0333 (6)	0.0342 (6)	0.0301 (6)	0.0046 (5)	0.0096 (5)	0.0081 (5)
N2A	0.0372 (7)	0.0298 (6)	0.0332 (6)	−0.0004 (5)	0.0060 (5)	0.0086 (5)
C1A	0.0417 (8)	0.0422 (9)	0.0399 (8)	0.0020 (7)	0.0123 (7)	0.0149 (7)
C2A	0.0452 (9)	0.0383 (8)	0.0305 (7)	0.0060 (7)	0.0124 (6)	0.0105 (6)
C3A	0.0591 (10)	0.0377 (9)	0.0374 (8)	0.0035 (7)	0.0081 (7)	0.0026 (7)
C4A	0.0716 (12)	0.0331 (8)	0.0468 (10)	0.0140 (8)	0.0095 (9)	0.0039 (7)
C5A	0.0532 (10)	0.0405 (9)	0.0366 (8)	0.0172 (7)	0.0085 (7)	0.0059 (7)
C6A	0.0491 (10)	0.0522 (10)	0.0404 (9)	0.0139 (8)	0.0073 (7)	0.0065 (8)
C7A	0.0456 (9)	0.0368 (8)	0.0326 (8)	0.0072 (7)	0.0123 (6)	0.0029 (6)
C8A	0.0468 (9)	0.0317 (8)	0.0384 (8)	0.0078 (6)	0.0138 (7)	0.0081 (6)
C9A	0.0312 (7)	0.0382 (8)	0.0332 (7)	0.0041 (6)	0.0081 (6)	0.0055 (6)
C10A	0.0354 (7)	0.0349 (8)	0.0368 (8)	0.0078 (6)	0.0065 (6)	0.0053 (6)
C11A	0.0315 (7)	0.0378 (8)	0.0375 (8)	0.0017 (6)	0.0105 (6)	0.0094 (6)
C12A	0.0374 (8)	0.0328 (7)	0.0335 (7)	0.0074 (6)	0.0114 (6)	0.0067 (6)
O1B	0.0434 (6)	0.0370 (6)	0.0553 (7)	0.0035 (5)	0.0167 (5)	0.0182 (5)
O2B	0.0420 (7)	0.0654 (9)	0.0692 (9)	−0.0174 (6)	0.0100 (6)	0.0172 (7)
O3B	0.0658 (9)	0.0635 (9)	0.0555 (8)	0.0021 (7)	0.0009 (7)	0.0353 (7)
O4B	0.0504 (7)	0.0568 (8)	0.0712 (9)	−0.0115 (6)	0.0075 (7)	0.0281 (7)
N1B	0.0471 (8)	0.0380 (7)	0.0393 (7)	0.0017 (6)	0.0096 (6)	0.0128 (6)
C1B	0.0330 (7)	0.0255 (7)	0.0334 (7)	0.0031 (5)	0.0107 (6)	−0.0002 (6)
C2B	0.0300 (7)	0.0373 (8)	0.0395 (8)	0.0008 (6)	0.0050 (6)	0.0015 (6)
C3B	0.0381 (8)	0.0400 (8)	0.0327 (8)	0.0077 (6)	0.0022 (6)	0.0080 (6)
C4B	0.0390 (8)	0.0286 (7)	0.0299 (7)	0.0032 (6)	0.0092 (6)	0.0056 (6)
C5B	0.0302 (7)	0.0327 (7)	0.0350 (8)	−0.0017 (6)	0.0032 (6)	0.0062 (6)
C6B	0.0338 (7)	0.0309 (7)	0.0338 (7)	0.0033 (6)	0.0027 (6)	0.0068 (6)
C7B	0.0367 (8)	0.0295 (7)	0.0436 (9)	0.0019 (6)	0.0170 (7)	0.0028 (6)
O1W	0.0347 (5)	0.0365 (6)	0.0422 (6)	−0.0020 (4)	0.0061 (5)	0.0073 (5)

### Geometric parameters (Å, º)

**Table:**

O1A—C5A	1.373 (2)	C9A—C10A	1.509 (2)
O1A—C6A	1.421 (2)	C10A—H10A	0.9900
O2A—C6A	1.431 (2)	C10A—H10B	0.9900
O2A—C7A	1.3802 (19)	C11A—H11A	0.9900
N1A—C1A	1.4619 (19)	C11A—H11B	0.9900
N1A—C9A	1.4617 (18)	C11A—C12A	1.511 (2)
N1A—C12A	1.4648 (18)	C12A—H12A	0.9900
N2A—H2AA	0.9422	C12A—H12B	0.9900
N2A—H2AB	0.9268	O1B—C7B	1.2622 (19)
N2A—C10A	1.4888 (19)	O2B—C7B	1.2400 (19)
N2A—C11A	1.4913 (18)	O3B—N1B	1.2192 (18)
C1A—H1AA	0.9900	O4B—N1B	1.2231 (18)
C1A—H1AB	0.9900	N1B—C4B	1.4693 (19)
C1A—C2A	1.509 (2)	C1B—C2B	1.393 (2)
C2A—C3A	1.384 (2)	C1B—C6B	1.388 (2)
C2A—C8A	1.408 (2)	C1B—C7B	1.516 (2)
C3A—H3A	0.9500	C2B—H2B	0.9500
C3A—C4A	1.395 (2)	C2B—C3B	1.387 (2)
C4A—H4A	0.9500	C3B—H3B	0.9500
C4A—C5A	1.366 (3)	C3B—C4B	1.379 (2)
C5A—C7A	1.379 (2)	C4B—C5B	1.379 (2)
C6A—H6AA	0.9900	C5B—H5B	0.9500
C6A—H6AB	0.9900	C5B—C6B	1.385 (2)
C7A—C8A	1.367 (2)	C6B—H6B	0.9500
C8A—H8A	0.9500	O1W—H1WA	0.8987
C9A—H9AA	0.9900	O1W—H1WB	0.9158
C9A—H9AB	0.9900		
			
C5A—O1A—C6A	106.07 (13)	C10A—C9A—H9AA	109.6
C7A—O2A—C6A	105.74 (13)	C10A—C9A—H9AB	109.6
C1A—N1A—C12A	111.33 (12)	N2A—C10A—C9A	109.95 (12)
C9A—N1A—C1A	109.81 (12)	N2A—C10A—H10A	109.7
C9A—N1A—C12A	108.99 (11)	N2A—C10A—H10B	109.7
H2AA—N2A—H2AB	107.3	C9A—C10A—H10A	109.7
C10A—N2A—H2AA	113.1	C9A—C10A—H10B	109.7
C10A—N2A—H2AB	113.7	H10A—C10A—H10B	108.2
C10A—N2A—C11A	110.93 (11)	N2A—C11A—H11A	109.7
C11A—N2A—H2AA	104.7	N2A—C11A—H11B	109.7
C11A—N2A—H2AB	106.6	N2A—C11A—C12A	109.91 (12)
N1A—C1A—H1AA	109.0	H11A—C11A—H11B	108.2
N1A—C1A—H1AB	109.0	C12A—C11A—H11A	109.7
N1A—C1A—C2A	112.76 (13)	C12A—C11A—H11B	109.7
H1AA—C1A—H1AB	107.8	N1A—C12A—C11A	110.19 (12)
C2A—C1A—H1AA	109.0	N1A—C12A—H12A	109.6
C2A—C1A—H1AB	109.0	N1A—C12A—H12B	109.6
C3A—C2A—C1A	120.29 (15)	C11A—C12A—H12A	109.6
C3A—C2A—C8A	119.60 (15)	C11A—C12A—H12B	109.6
C8A—C2A—C1A	120.09 (14)	H12A—C12A—H12B	108.1
C2A—C3A—H3A	118.8	O3B—N1B—O4B	123.48 (14)
C2A—C3A—C4A	122.45 (17)	O3B—N1B—C4B	117.98 (14)
C4A—C3A—H3A	118.8	O4B—N1B—C4B	118.52 (13)
C3A—C4A—H4A	121.6	C2B—C1B—C7B	119.39 (13)
C5A—C4A—C3A	116.74 (16)	C6B—C1B—C2B	119.79 (14)
C5A—C4A—H4A	121.6	C6B—C1B—C7B	120.82 (13)
O1A—C5A—C7A	110.07 (15)	C1B—C2B—H2B	119.6
C4A—C5A—O1A	128.41 (16)	C3B—C2B—C1B	120.75 (14)
C4A—C5A—C7A	121.52 (16)	C3B—C2B—H2B	119.6
O1A—C6A—O2A	108.35 (14)	C2B—C3B—H3B	121.1
O1A—C6A—H6AA	110.0	C4B—C3B—C2B	117.81 (14)
O1A—C6A—H6AB	110.0	C4B—C3B—H3B	121.1
O2A—C6A—H6AA	110.0	C3B—C4B—N1B	119.33 (13)
O2A—C6A—H6AB	110.0	C3B—C4B—C5B	122.89 (14)
H6AA—C6A—H6AB	108.4	C5B—C4B—N1B	117.78 (13)
C5A—C7A—O2A	109.55 (14)	C4B—C5B—H5B	120.7
C8A—C7A—O2A	127.90 (14)	C4B—C5B—C6B	118.61 (13)
C8A—C7A—C5A	122.54 (15)	C6B—C5B—H5B	120.7
C2A—C8A—H8A	121.4	C1B—C6B—H6B	119.9
C7A—C8A—C2A	117.14 (14)	C5B—C6B—C1B	120.13 (13)
C7A—C8A—H8A	121.4	C5B—C6B—H6B	119.9
N1A—C9A—H9AA	109.6	O1B—C7B—C1B	117.18 (13)
N1A—C9A—H9AB	109.6	O2B—C7B—O1B	125.94 (15)
N1A—C9A—C10A	110.19 (12)	O2B—C7B—C1B	116.88 (14)
H9AA—C9A—H9AB	108.1	H1WA—O1W—H1WB	106.6
			
O1A—C5A—C7A—O2A	0.01 (19)	C9A—N1A—C1A—C2A	−173.67 (12)
O1A—C5A—C7A—C8A	179.33 (15)	C9A—N1A—C12A—C11A	61.80 (15)
O2A—C7A—C8A—C2A	179.03 (14)	C10A—N2A—C11A—C12A	55.02 (16)
N1A—C1A—C2A—C3A	−135.98 (16)	C11A—N2A—C10A—C9A	−55.19 (16)
N1A—C1A—C2A—C8A	45.6 (2)	C12A—N1A—C1A—C2A	65.54 (16)
N1A—C9A—C10A—N2A	58.74 (16)	C12A—N1A—C9A—C10A	−61.96 (15)
N2A—C11A—C12A—N1A	−58.34 (16)	O3B—N1B—C4B—C3B	3.6 (2)
C1A—N1A—C9A—C10A	175.85 (12)	O3B—N1B—C4B—C5B	−176.66 (15)
C1A—N1A—C12A—C11A	−176.93 (12)	O4B—N1B—C4B—C3B	−175.27 (15)
C1A—C2A—C3A—C4A	−178.95 (15)	O4B—N1B—C4B—C5B	4.5 (2)
C1A—C2A—C8A—C7A	178.88 (13)	N1B—C4B—C5B—C6B	−178.38 (13)
C2A—C3A—C4A—C5A	0.3 (3)	C1B—C2B—C3B—C4B	−1.4 (2)
C3A—C2A—C8A—C7A	0.5 (2)	C2B—C1B—C6B—C5B	−0.6 (2)
C3A—C4A—C5A—O1A	−179.27 (16)	C2B—C1B—C7B—O1B	176.26 (13)
C3A—C4A—C5A—C7A	0.0 (3)	C2B—C1B—C7B—O2B	−4.0 (2)
C4A—C5A—C7A—O2A	−179.42 (16)	C2B—C3B—C4B—N1B	179.53 (13)
C4A—C5A—C7A—C8A	−0.1 (3)	C2B—C3B—C4B—C5B	−0.2 (2)
C5A—O1A—C6A—O2A	−4.64 (19)	C3B—C4B—C5B—C6B	1.3 (2)
C5A—C7A—C8A—C2A	−0.2 (2)	C4B—C5B—C6B—C1B	−0.9 (2)
C6A—O1A—C5A—C4A	−177.74 (18)	C6B—C1B—C2B—C3B	1.8 (2)
C6A—O1A—C5A—C7A	2.89 (19)	C6B—C1B—C7B—O1B	−3.2 (2)
C6A—O2A—C7A—C5A	−2.88 (18)	C6B—C1B—C7B—O2B	176.51 (14)
C6A—O2A—C7A—C8A	177.85 (16)	C7B—C1B—C2B—C3B	−177.71 (13)
C7A—O2A—C6A—O1A	4.63 (19)	C7B—C1B—C6B—C5B	178.89 (13)
C8A—C2A—C3A—C4A	−0.5 (3)		

### Hydrogen-bond geometry (Å, º)

**Table:**

*D*—H···*A*	*D*—H	H···*A*	*D*···*A*	*D*—H···*A*
N2*A*—H2*AA*···O1*W*^i^	0.94	1.84	2.7800 (16)	172
N2*A*—H2*AB*···O1*B*^ii^	0.93	1.80	2.7262 (16)	175
C9*A*—H9*AA*···O2*A*^iii^	0.99	2.58	3.3260 (19)	132
C10*A*—H10*A*···O1*W*^iv^	0.99	2.51	3.2833 (19)	135
O1*W*—H1*WA*···O2*B*^v^	0.90	1.76	2.6526 (16)	170
O1*W*—H1*WB*···O1*B*^ii^	0.92	1.90	2.7867 (16)	163

Symmetry codes: (i) −*x*, −*y*+1, −*z*; (ii) −*x*+1, −*y*+1, −*z*; (iii) −*x*, −*y*+1, −*z*+1; (iv) *x*−1, *y*, *z*; (v) −*x*+2, −*y*+1, −*z*.
